# Multiple Information Sources and Consequences of Conflicting Information About Medicine Use During Pregnancy: A Multinational Internet-Based Survey

**DOI:** 10.2196/jmir.2939

**Published:** 2014-02-20

**Authors:** Katri Hämeen-Anttila, Hedvig Nordeng, Esa Kokki, Johanna Jyrkkä, Angela Lupattelli, Kirsti Vainio, Hannes Enlund

**Affiliations:** ^1^Assessment of PharmacotherapiesFinnish Medicines AgencyFimeaFinland; ^2^School of PharmacyUniversity of OsloOsloNorway; ^3^School of PharmacyFaculty of Health SciencesUniversity of Eastern FinlandKuopioFinland

**Keywords:** pharmaceutical preparations, pregnancy, access to information, information seeking behavior, information dissemination, questionnaires, Internet, international

## Abstract

**Background:**

A wide variety of information sources on medicines is available for pregnant women. When using multiple information sources, there is the risk that information will vary or even conflict.

**Objective:**

The objective of this multinational study was to analyze the extent to which pregnant women use multiple information sources and the consequences of conflicting information, and to investigate which maternal sociodemographic, lifestyle, and medical factors were associated with these objectives.

**Methods:**

An anonymous Internet-based questionnaire was made accessible during a period of 2 months, on 1 to 4 Internet websites used by pregnant women in 5 regions (Eastern Europe, Western Europe, Northern Europe, Americas, Australia). A total of 7092 responses were obtained (n=5090 pregnant women; n=2002 women with a child younger than 25 weeks). Descriptive statistics and logistic regression analysis were used.

**Results:**

Of the respondents who stated that they needed information, 16.16% (655/4054) used one information source and 83.69% (3393/4054) used multiple information sources. Of respondents who used more than one information source, 22.62% (759/3355) stated that the information was conflicted. According to multivariate logistic regression analysis, factors significantly associated with experiencing conflict in medicine information included being a mother (OR 1.32, 95% CI 1.11-1.58), having university (OR 1.33, 95% CI 1.09-1.63) or other education (OR 1.49, 95% CI 1.09-2.03), residing in Eastern Europe (OR 1.52, 95% CI 1.22-1.89) or Australia (OR 2.28, 95% CI 1.42-3.67), use of 3 (OR 1.29, 95% CI 1.04-1.60) or >4 information sources (OR 1.82, 95% CI 1.49-2.23), and having ≥2 chronic diseases (OR 1.49, 95% CI 1.18-1.89). Because of conflicting information, 43.61% (331/759) decided not to use medication during pregnancy, 30.30% (230/759) sought a new information source, 32.67% (248/759) chose to rely on one source and ignore the conflicting one, 25.03% (190/759) became anxious, and 2.64% (20/759) did nothing. Factors significantly associated with not using medication as a consequence of conflicting information were being pregnant (OR 1.75, 95% CI 1.28-2.41) or experiencing 3-4 health disorders (OR 1.99, 95% CI 1.10-3.58). Women with no chronic diseases were more likely not to take medicines than women with ≥2 chronic diseases (OR 2.22, 95% CI 1.47-3.45). Factors significantly associated with becoming anxious were >4 information sources (OR 2.67, 95% CI 1.70-4.18) and residing in Eastern Europe (OR 0.57, 95% CI 0.36-0.90).

**Conclusions:**

Almost all the pregnant women used multiple information sources when seeking information on taking medicines during pregnancy and one-fifth obtained conflicting information, leading to anxiety and the decision not to use the medication. Regional, educational, and chronic disease characteristics were associated with experiencing conflicting information and influenced the decision not to use medication or increased anxiety. Accurate and uniform teratology information should be made more available to the public.

## Introduction

Use of both prescription medicines and over-the-counter medicines during pregnancy is common [1-3]. Pregnant women tend to be cautious about using medication and tend to have unrealistic perceptions of drug-related teratogenic risks [4,5]. This may result in nonadherence to therapy or even to the unjustified termination of a pregnancy [6,7]. Relatively few studies focus on adherence to drug therapy during pregnancy [8]. Those that do have shown that the overall adherence rate during pregnancy is approximately 40% [9-11]. Adherence to chronic medication seems higher than to short-term medication or symptomatic treatment [10], but the opposite has also been reported in treatment of asthma [12] and depression [13]. Poor adherence among pregnant women is typically seen as reduction of the dose, total discontinuation of medication [9,14,15], or forgetting to take the medication [16,17]. Conflicting information from different sources may add to the uncertainty about whether or not to use medication [5,13].

Pregnant women use health care professionals, most commonly physicians, but also pharmacists and nurses, as primary information sources [5,18-20]. The Internet is also a widely used information source [5,18,19]. A recent multinational study showed that 70% of the responding pregnant women who indicated a need for information used the Internet as an information source, varying from 44% in Canada to 90% in Russia [19]. Other information sources include patient information leaflets, family and friends, drug information centers, books, and magazines.

This indicates the existence of a wide variety of formal and informal information sources, which pregnant women may consult simultaneously. In fact, Henry and Crowther [20] reported in their review that 4 information sources are used on average, with one-quarter of respondents consulting more than 5 sources (range 0-11 information sources among different studies). With an increasing number of information sources, there is also an increased risk that information will vary or even conflict. In a Norwegian study of pregnant women who used several information sources, 25% reported the presence of conflicting information between sources [5]. Thus, it may be hypothesized that conflicting information increases the possibility that a medicine will not be used during pregnancy even if it is safe and important to the pregnant woman and the unborn child.

This multinational survey aims to identify the extent to which pregnant women use multiple information sources and the consequences of the presence of conflicting information. Furthermore, it aims to investigate which maternal sociodemographic, lifestyle, and medical factors are associated with these objectives.

## Methods

### Study Design

This study forms part of a multinational Internet-based survey on medication use in pregnancy, which investigates medicine use, health disorders, and chronic diseases during pregnancy; perceptions of risks and attitudes toward using medicines; and needs for information [11]. The survey was conducted in 5 regions: Eastern Europe (Croatia, Poland, Russia, Serbia, Slovenia), Western Europe (Austria, France, Italy, the Netherlands, Switzerland, United Kingdom), Northern Europe (Finland, Iceland, Norway, Sweden), Americas (Canada, United States, South America), and Australia.

An anonymous self-completed Internet-based questionnaire [21] was posted on 1 to 4 Internet websites used by pregnant women in different countries. Originally developed at the University of Oslo [5], the questionnaire was translated into the respective languages of the participating countries. The questionnaire was piloted in 4 countries (pilot responses are not included in the study). Only minor modifications were made. During the pilot, the usability and technical functionality of the electronic questionnaire were also tested. Adaptive questioning was used to reduce the number and complexity of the questions. The questionnaire included various topics concerning medicine use and health during pregnancy, as well as attitudes toward using medicines in general and during pregnancy. In this study, data were used that had been gathered based on responses to questions concerning the need for information and reported information sources.

The questionnaire was accessible during a period of 2 months in each country, from October 1, 2011 to February 29, 2012. Pregnant women and women with a child younger than 25 weeks old were eligible to participate in this study. Respondents were advised to answer questions related to their current or latest pregnancy. The participants were also asked to read the study description, along with the study objectives and other relevant information, before being given access to the online questionnaire. Reading the study description and confirming a wish to participate were considered the equivalent of giving informed consent. Thereafter, the woman was given access to the online questionnaire. No personal identifiable information was collected. Ethical approval was sought and granted from the Norwegian Regional Ethics Committee.

In each country, the study population was compared to the birthing population by using national or population-based statistics to evaluate external validity [19]. Overall, the mean age of the study populations in each country was close to the mean age of the target populations. Respondents were somewhat better educated than average women, and the percentage of primiparity was higher among respondents than among most national populations. Of the respondents, 57.16% (4054/7092) reported a need for information on medicines during their pregnancy [19]. The most commonly used information sources were health care professionals (physicians: 73.14%, 2965/4054; pharmacy personnel: 46.18%, 1872/4054; midwifes or nurses: 32.51%, 1318/4054) and the Internet (59.50%, 2412/4054). A detailed description of information needs and the information sources used is given elsewhere [19].

### Main Outcome Measures

A list of commonly used sources was given to explore the number and type of information sources used. Respondents were also given the opportunity to mention additional sources. The basis for this list was taken from a previous study by the authors [5] and adjusted in relation to the information sources available in the participating countries. These were further categorized into formal information sources (including physicians, pharmacists, midwifes, nurses, drug handbooks, information leaflets, and drug information centers) and informal information sources (including Internet, family and friends, magazines, media, books, and herbal shop personnel). Drug information centers refer to medicine information services (also known drug information call centers) where people can call or otherwise contact health care professionals and inquire about medicines.

The need for information was assessed with the question, “Did you need information on medicines during the course of your pregnancy?” Respondents who indicated a need for information (n=4054) were further asked if the information they had found from various sources was uniform. The respondents could choose from 4 responses: (1) yes, completely similar; (2) yes, on the whole (only the wording or level of detail was somewhat different); (3) no, part of the information was different; and (4) no, the information was completely contradictory. These answers were further classified into the categories “yes” (2 former categories) and “no” (2 latter categories).

If the respondent’s answers fell into any of the “no” categories, she was further asked about her subsequent actions with the question: “If there were discrepancies among the sources, what did this mean to you? (You may tick more than one answer).” The 5 possible responses were: (1) nothing, (2) I became anxious, (3) I decided not to use the medication, (4) I sought a new information source, and (5) I chose to rely on one source only, and to ignore the conflicting one.

### Background Variables

The following background variables were used: the status of the women (pregnant or had given birth at the time of the study), age, parity, marital status, educational level, region of residence, number of chronic diseases, experienced health disorders, and number of information sources used.

### Statistical Analysis

We used SPSS version 20 (IBM Corp, Armonk, NY, USA) for statistical analyses. Cross-tabulation and Pearson’s chi-square test were used as univariate analysis when analyzing categorical variables. A *P* value of <.05 was considered to be statistically significant. For multivariate analysis, logistic regression analysis including odds ratios (OR) and 95% confidence intervals (95% CIs) were used when measuring the association of maternal sociodemographics and lifestyle characteristics with the experience of conflicting information and with the consequences of the presence of conflicting information during pregnancy. The stepwise method (forward conditional) was used to select the variables in the final model. Multivariate analyses were conducted for the consequences “did not use medication” and “became anxious.” The Hosmer and Lemeshow test [22] was used to assess goodness-of-fit of the final multivariate models and the models were found robust (*P* values >.05).

## Results

### Summary

A total of 9615 women in various countries accessed the online open survey. Of these women, 9483 (98.63%) agreed to participate and filled in the questionnaire. Of the related responses, 7092 of 9483 (74.79%) were eligible for this study, including 5090 (53.67%) responses from pregnant women and 2002 (21.11%) from women with a child younger than 25 weeks.

### Information Sources on Medicines

**Figure 1:**
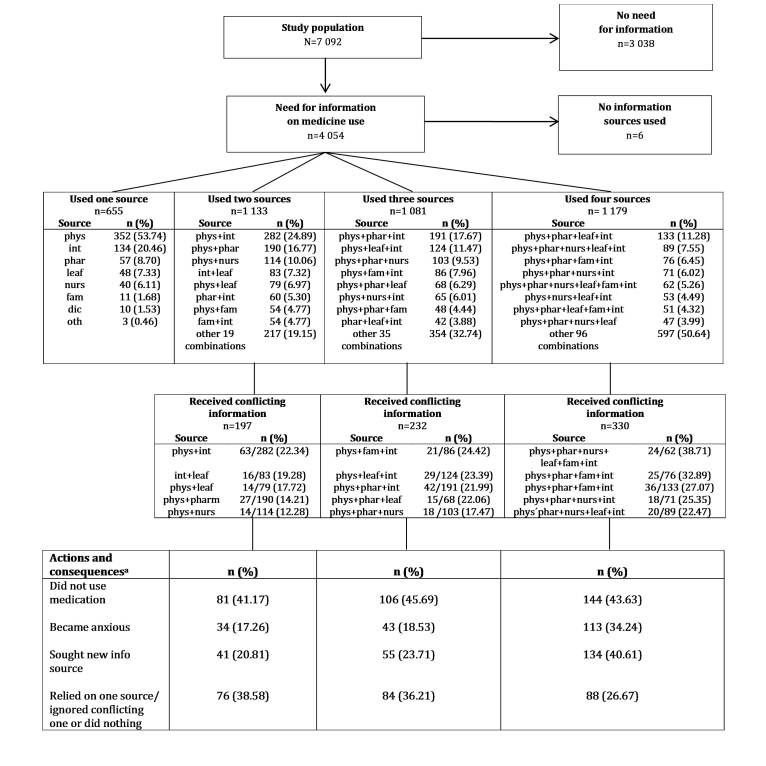
Need for information on medicine use including actions and consequences ensuing from conflicting information. Phys: physicians; phar: pharmacists; nurs: nurses or midwives; leaf: patient information leaflets; dic: drug information centers; int: Internet; fam: family or friends.

On average, the respondents used 3 different information sources on medicines (range 0-8). Of these, 16.16% (655/4054) used 1 source, 27.95% (1133/4054) used 2 sources, 26.66% (1081/4054) used 3 sources, and 29.08% (1179/4054) used 4 or more different information sources (Figure 1). A physician was used as the sole information source by 53.74% (352/655) of the women who used 1 information source, followed by the Internet (20.46%, 134/655) and a pharmacist (8.70%, 57/655) (Figure 1). Physicians (77.01%, 3613/3393), the Internet (67.14%, 2278/3393), and pharmacists (53.49%, 1815/3393) also featured most often among the combinations of various information sources. Other sources included nurses or midwives; patient information leaflets; drug information centers; family or friends; books, magazines, or media; and herbal shops.

Of the women needing information, 94.44% (3829/4054) used formal information sources (including physicians, pharmacists, midwifes, nurses, drug handbooks, patient information leaflets, and drug information centers) and 67.04% (2718/4054) used informal information sources (including the Internet, family and friends, magazines, media, books, and herbal shop personnel). The Internet was used by 59.50% (2412/4054) of women needing information. Both formal and informal information sources were used by 61.64% (2499/4054).

### Conflicting Information From Different Sources

Of the respondents who used more than 1 information source, 22.62% (759/3355) indicated that information was conflicting. The proportion of women receiving conflicting information increased along with the number of sources. This varied from 2 information sources, of which 17.39% (197/1133) consisted of conflicting information, to 4 or more information sources, of which 27.99% (330/1179) contained conflicting information (Figure 1).

The experience of conflicting information was greatest among the mothers who had university education or other education (Table 1). Furthermore, high numbers of information sources and high numbers of chronic diseases were associated with the experienced information conflicts. There were also regional differences: women from Eastern Europe and Australia reporting experiencing conflicting information more often than women in other regions.

**Table 1:** Univariate and multivariate analysis of factors associated with conflict of information (n=3355).

Variable		n	Information type, n (%)		Contradictory information			
			Similar information	Contradictory information	Univariate logistic regression		Multivariate logistic regression^a^	
					OR (95% CI)	*P*	OR (95% CI)	*P*
**Status of the woman**						.001		.002
	Pregnant	2379	1878 (78.9)	501 (21.1)	1.00		1.00	
	Mother	976	718 (73.6)	258 (26.4)	1.35 (1.13-1.60)		1.32 (1.11-1.58)	
**Age (years)**						.08	—	
	≤24	615	496 (80.7)	119 (19.3)	0.77 (0.62-0.97)			
	25-34	2173	1659 (76.3)	514 (23.7)	1.00			
	≥35	531	412 (77.6)	119 (22.4)	0.93 (0.74-1.17)			
**Parity**						.21	—	
	Primiparous	1928	1507 (78.2)	421 (21.8)	1.00			
	≥1 previous children	1427	1089 (76.3)	338 (23.7)	1.11 (0.94-1.31)			
**Marital status**						.15	—	
	Married or cohabitant	3149	2445 (77.6)	704 (22.4)	1.00			
	Other	206	151 (73.3)	55 (26.7)	1.27 (0.92-1.74)			
**Education level**						.003		.02
	Primary school	153	124 (81.0)	29 (19.0)	1.03 (0.66-1.59)		1.02 (0.65-1.62)	
	High/secondary school	884	720 (81.4)	164 (18.6)	1.00		1.00	
	University	1990	1507 (75.7)	483 (24.3)	1.41 (1.15-1.72)		1.33 (1.09-1.63)	
	Other Education	328	245 (74.7)	83 (25.3)	1.49 (1.10-2.01)		1.49 (1.09-2.03)	
**Region of residence**						<.001		<.001
	Western Europe	1030	838 (81.4)	192 (18.6)	1.00		1.00	
	Eastern Europe	919	676 (73.6)	243 (26.4)	1.57 (1.27-1.95)		1.52 (1.22-1.89)	
	Northern Europe	1046	817 (78.1)	229 (21.9)	1.22 (0.99-1.52)		1.15 (0.92-1.44)	
	Americas	274	211 (77.0)	63 (23.0)	1.30 (0.94-1.80)		1.28 (0.92-1.77)	
	Australia	86	54 (62.8)	32 (37.2)	2.59 (1.63-4.11)		2.28 (1.42-3.67)	
**Experienced health disorders**						.63	—	
	0-2	310	246 (79.4)	64 (20.6)	1.00			
	3-4	1003	770 (76.8)	233 (23.2)	0.90 (0.67-1.20)			
	≥5	2028	1572 (77.5)	456 (22.5)	1.04 (0.87-1.25)			
**Number of chronic diseases**						<.001		.002
	0	2294	1816 (79.2)	478 (20.8)	1.00		1.00	
	1	592	446 (75.3)	146 (24.7)	1.24 (1.01-1.54)		1.20 (0.96-1.49)	
	≥2	469	334 (71.2)	135 (28.8)	1.54 (1.23-1.92)		1.49 (1.18-1.89)	
**Multiple information sources**						<.001		<.001
	2 sources	1112	915 (82.3)	197 (17.7)	1.00		1.00	
	3 sources	1071	839 (78.3)	232 (21.7)	1.28 (1.04-1.59)		1.29 (1.04-1.60)	
	≥4 sources	1172	842 (71.8)	330 (28.2)	1.82 (1.49-2.22)		1.82 (1.49-2.23)	

^a^Adjusted with the variables shown (n=3305).

### Responses to Conflicting Information on Medicines

Almost half (43.6%, 332/759) of women decided not to use medication because of conflicting information; 30.3% (230/759) sought a new information source; 32.7% (248/759) chose to rely on one source and ignore the conflicting one; 25.0% (190/759) became anxious; and 2.6% (20/759) did nothing (Table 2).

According to the multivariate analysis, pregnant women (47.9%, 240/501) more often than mothers (35.3%, 91/258) reported not using medication as a consequence of conflicting information when adjusted for other variables (OR 1.75, 95% CI 1.28-2.41). This was also the case for women who had experienced 3-4 health disorders (50.2%, 117/233) compared to women with experience of 0-2 health disorders (35.9%, 23/64; OR 1.99, 95% CI 1.10-3.58). Moreover, women with no chronic diseases (48.6%, 228/478) were more likely to state that they did not use medicines than women with 2 or more chronic diseases (28.9%, 39/135; OR 2.22, 95% CI 1.47-3.45). No other significant associations were found.

Multivariate analysis also showed that women who used 4 or more information sources (34.2%, 113/330) reported becoming anxious as a consequence of conflicting information more often than women who used 2 information sources (17.2%, 34/197; OR 2.67, 95% CI 1.70-4.18) after adjustment for other variables. Furthermore, women from Eastern Europe (19.7%, 48/243) were less likely to report becoming anxious than women from Western Europe (28.1%, 54/192; OR 0.57, 95% CI 0.36-0.90). Again, no other significant associations were found.

**Table 2:** Consequences of discrepancies between different information sources by background variables (n=759).

Variable		n	Consequence,^a^ n (%)			
			Did not use medication	Became anxious	Sought a new information source	Relied on one source and ignored conflicting one, or did nothing
**Status of the woman**						
	Pregnant	501	240 (47.9)	118 (23.6)	135 (26.9)	161 (32.1)
	Mother	258	91 (35.3)	72 (27.9)	95 (36.8)	87 (33.7)
**Age (years)**						
	≤24	119	63 (52.9)	28 (23.5)	36 (30.3)	30 (25.2)
	25-34	514	217 (42.2)	132 (25.7)	163 (31.7)	173 (33.7)
	≥35	119	49 (41.2)	27 (22.7)	31 (26.0)	41 (34.5)
**Parity**						
	Primiparous	421	185 (43.9)	105 (24.9)	129 (30.6)	135 (32.1)
	≥1 previous children	338	146 (43.2)	85 (25.1)	101 (29.9)	113 (33.4)
**Marital status**						
	Married or cohabitant	704	308 (43.7)	170 (24.1)	215 (30.6)	232 (32.9)
	Other	55	23 (41.8)	20 (36.4)	15 (27.3)	16 (29.1)
**Education level**						
	Primary school	29	11 (37.9)	5 (17.2)	7 (24.1)	14 (48.3)
	High/secondary school	164	81 (49.4)	46 (28.0)	27 (16.5)	54 (32.9)
	University	483	206 (42.6)	110 (22.8)	168 (34.8)	157 (32.5)
	Other education	83	33 (39.7)	29 (34.9)	28 (33.7)	21 (25.3)
**Region of residence**						
	Western Europe	192	84 (43.7)	54 (28.1)	43 (22.4)	61 (31.8)
	Eastern Europe	243	118 (48.6)	48 (19.7)	87 (35.8)	67 (27.6)
	Northern Europe	229	95 (41.5)	58 (25.3)	65 (28.4)	91 (39.7)
	Americas	63	25 (39.7)	21 (33.3)	21 (33.3)	19 (30.2)
	Australia	32	9 (28.1)	9 (28.1)	14 (43.7)	10 (31.2)
**Experienced health disorders**						
	0-2	64	23 (35.9)	11 (17.2)	20 (31.2)	19 (29.7)
	3-4	233	117 (50.2)	50 (21.4)	20 (8.6)	75 (32.2)
	≥5	456	189 (41.4)	128 (28.1)	137 (30.0)	153 (33.6)
**Number of chronic diseases**						
	0	478	228 (47.7)	114 (23.8)	122 (25.5)	147 (30.7)
	1	146	64 (43.8)	36 (24.6)	56 (38.3)	44 (30.1)
	≥2	135	39 (28.9)	40 (29.6)	52 (38.5)	57 (42.2)
**Multiple information sources**						
	2	197	81 (41.1)	34 (17.2)	41 (20.8)	76 (38.6)
	3	232	106 (45.7)	43 (18.5)	55 (23.7)	84 (36.2)
	≥4	330	144 (43.6)	113 (34.2)	134 (40.6)	88 (26.7)

^a^Respondents could choose more than 1 answer.

## Discussion

### Principal Findings

According to this study, most (83.69%, 3393/4054) pregnant women reported used multiple information sources when seeking information on medicine use during pregnancy. Of these, approximately one-fifth (18.7%, 759/4054) reported that information received from multiple sources was conflicting. Experiencing conflicting information was associated with being a mother (compared to being pregnant), having university or other education, having chronic diseases, and using multiple information sources. Furthermore, regional differences were found. As a consequence, substantial numbers of women who received conflicting information decided not to use their medication. Increased anxiety was also common. These results confirm earlier results recorded in studies of adherence problems [8,12-15].

According to our results and the results of previous studies, pregnant women actively seek information on medicines and health from various information sources [5,18-20]. To our knowledge, our study is the first to reveal the extent and wide variety of combinations of information sources used by pregnant women from 5 regions of the world involved in this study. However, in each case, we do not know which source came first, in which order the information was sought, or the actual content of the information received. There is evidence of a lack of consistency between information on drug safety during pregnancy based on different information sources [23-25]. For example, according to the study by Frost Widnes and Schjøtt [24], the Norwegian Compendium on Product Monographs gives advice which is more restrictive than that given by drug information centers. Furthermore, patient information leaflets have been shown to include varying information concerning medicine use during pregnancy when different brand names of the same active substance are compared [23]. Thus, it is not surprising that we found that the number of information sources was associated with the experience of conflict in medical information.

Importantly, pregnant women may find safe lists for medications that can be taken during pregnancy from the Internet that have no basis in evidence [25]. This suggests a clear risk of unnecessary use of medicines, misunderstandings, and groundless anxiety that could be avoided by the deployment of relevant, uniform, and accurate information. It has been argued that teratology information services (TIS) are effective in teratology information knowledge transfer by using evidence-based information expressed in lay language [26-28]. These TIS exist all over North America, in Australia, and many European countries.

The Internet is commonly used as a supplementary information source, before or after contacting a health care professional [29-31]. Women not only seek factual knowledge from the Internet, but also look for emotional support and encouragement, especially from other women in the same life situation [29,32]. Women determine which websites they can trust based on methods that include repetition of the same facts on various sites [29-31]. This leads to confusion about what information to trust when it proves to be inconsistent among different sites. Despite this, most women do not consult health care professionals about the health information they have retrieved [30,31]. Every health care professional, including physicians, nurses, midwifes, and pharmacists, should be active in asking pregnant women whether or not they have sought information on the Internet and about where and what kind of information they have found and if the information found has raised any questions. In addition, the basic education of all health care professionals should include information on reliable online information sources, targeted to medicine users as well as professionals, and about how such information sources should be discussed with the medicine user.

### Limitations

This study has several limitations that should be considered when interpreting the results. Because the questionnaire was only available through the Internet, we are unable to calculate a conventional response rate. This made a comparison with the birthing population of each of the participating countries necessary to determine representativeness. However, such comparisons could not be made for South American countries because birthing population reports were not available. Furthermore, based on previous epidemiological studies using Web-based recruitment methods, a reasonable level of validity can be expected [33-35]. It should also be noted that we do not know precisely which websites the respondents used and because of the cross-sectional study design, neither do we know which information source was consulted first (eg, whether the Internet was used as the first point of enquiry or whether the women searched it for a second opinion after visiting a health care professional). Furthermore, education was categorized in the questionnaire including a possibility to choose “other education.” This is why we do not know what kind of education this includes.

The study population is fairly representative of the target populations of each participating country [19]. However, respondents were somewhat better educated and the study population had more primiparous women than the target population. Better-educated women tend to seek information and use multiple information sources [36,37], which may result in overestimating the need for information and use of multiple sources. It might also be assumed that women with medical problems or using medicines during pregnancy were more likely than others to respond to the survey.

### Conclusions

Almost all the pregnant women who needed information used multiple information sources when seeking information on medicines during pregnancy. One-fifth of the respondents had obtained conflicting information on medicine use during pregnancy. Being a mother, having university education, the number of chronic diseases, and number of information sources consulted was associated with the experience of conflicting information. Furthermore, region of residence in Eastern Europe or Australia was also associated with this experience.

Conflicting information often lead to anxiety and the decision not to use medication; pregnant women with 3-4 experienced health disorders but no chronic diseases reported not using medication as a consequence of conflicting information more often than others.

Action needs to be taken to make accurate and uniform teratology information more available to the public, such as ensuring that basic education of health care professionals includes medicine use during pregnancy. It is also important to increase the availability of reliable information (eg, TIS websites). Health care professionals should actively ask pregnant women about the information they have found from various sources and discuss this information with them.

## Abbreviations

TIS: teratology information services
